# GRK5‐mediated inflammation and fibrosis exert cardioprotective effects during the acute phase of myocardial infarction

**DOI:** 10.1002/2211-5463.13551

**Published:** 2023-01-20

**Authors:** Akiomi Nagasaka, Tsuyoshi Terawaki, Makoto Noda, Miyuki Takashima, Mika Fujino, Yuto Yamauchi, Shigeki Arawaka, Takeo Kato, Michio Nakaya

**Affiliations:** ^1^ Department of Pharmacology and Toxicology, Graduate School of Pharmaceutical Sciences Kyushu University Fukuoka Japan; ^2^ Department of Disease control, Graduate School of Pharmaceutical Sciences Kyushu University Fukuoka Japan; ^3^ Division of Neurology, Department of Internal Medicine IV Osaka Medical College Japan; ^4^ Division of Neurology and Clinical Neuroscience, Department of Internal Medicine III Yamagata University School of Medicine Japan; ^5^ AMED‐PRIME Japan Agency for Medical Research and Development Tokyo Japan

**Keywords:** fibroblast, fibrosis, G‐protein‐coupled receptor kinase, GRK5, myocardial infarction, myofibroblast

## Abstract

During myocardial infarction (MI), cardiac cells at the infarcted area undergo cell death. In response, cardiac myofibroblasts, which are mainly differentiated from resident fibroblasts upon inflammation, produce extracellular matrix proteins such as collagen to fill the damaged areas of the heart to prevent cardiac rupture. In this study, we identified a cardioprotective role of G‐protein‐coupled receptor kinase 5 (GRK5) in MI. GRK5 expression was found to increase in the mouse heart after MI and was highly expressed in cardiac fibroblasts/myofibroblasts. In fibroblasts/myofibroblasts, GRK5 promoted the expression of inflammation‐related genes through nuclear factor‐κB activation, leading to an increase in the expression levels of fibrosis‐related genes. Bone marrow transfer experiments confirmed that GRK5 in fibroblasts/myofibroblasts, but not in infiltrated macrophages in the infarcted area, is mainly responsible for GRK5‐mediated inflammation in infarcted hearts. In addition, inflammation and fibrosis at the infarcted area were significantly suppressed in GRK5 knockout mice, resulting in increased mortality compared with that in wild‐type mice. These data indicate that GRK5 in cardiac fibroblasts/myofibroblasts promotes inflammation and fibrosis to ameliorate the damage after MI.

## Graphical abstract

G‐protein‐coupled receptor kinase 5 (GRK5) is highly expressed in cardiac myofibroblasts and initiates inflammation through nuclear factor‐κB activation, leading to an increase in the expression levels of fibrosis‐related genes in the acute phase of myocardial infarction. Thus, GRK5 in cardiac myofibroblasts is an important molecule that promotes inflammation and fibrosis to ameliorate the damage after myocardial infarction.

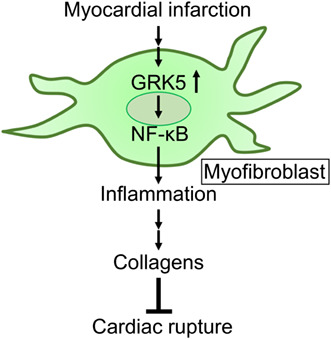

**Abbreviations**
COL1A1: collagen 1a1
CTGF: connective tissue growth factor
DMEM: Dulbecco's modified Eagle medium
FBS: fetal bovine serum
GAPDH: glyceraldehyde 3‐phosphate dehydrogenase
GPCR: G‐protein‐coupled receptor
GRK: G‐protein‐coupled receptor kinase
HDAC5: histone deacetylase 5
IκBα: nuclear factor κ light polypeptide gene enhancer in B‐cells inhibitor, α
KO: knockout
MI: myocardial infarction
NF‐κB: nuclear factor κB
PBS: phosphate‐buffered saline
PDGFR‐α: platelet‐derived growth factor receptor α
RT‐PCR: reverse transcription polymerase chain reaction
TGF‐β: transforming growth factor‐β
TNF‐α: tumor necrosis factor‐α
WT: wild‐type
α‐SMA: α‐smooth muscle actin

Myocardial infarction (MI) is caused by occlusion of the coronary artery in the heart and is known to result in high mortality [1]. When MI occurs, cardiomyocytes that are no longer supplied with oxygen and nutrients undergo cell death due to the obstruction of blood vessels. The dead cardiomyocytes in the infarcted area release damage‐associated molecular patterns, molecules that stimulate various hematopoietic cells such as neutrophils, macrophages, and fibroblasts. These cells produce inflammatory cytokines and chemokines, resulting in the promotion of inflammation [2]. Subsequently, these cells acquire an anti‐inflammatory phenotype, secreting anti‐inflammatory cytokines such as transforming growth factor (TGF)‐β [3], which promotes the differentiation of myofibroblasts from various precursor cell types. Thus, myofibroblasts synthesize excessive extracellular matrix proteins, resulting in fibrosis. Fibrosis is an important process that prevents ventricular wall rupture by replacing the damaged areas of heart tissue [4, 5]. Hence, identification of molecules that regulate inflammation and fibrosis after MI could be used to establish novel therapeutic strategies against MI.

G‐protein‐coupled receptor kinases (GRKs) phosphorylate activated G‐protein‐coupled receptors (GPCRs), bind with their agonists, and lead to their desensitization [6]. G‐protein‐coupled receptor kinases consist of seven homologs (GRK1–7). Recently, it was reported that GRKs could phosphorylate non‐GPCRs and regulate various intracellular pathways [7]. The role of GRK5 was reported in cardiac hypertrophy using transverse aortic constriction [8, 9]. These reports concluded that GRK5 expressed in cardiomyocytes acts as a histone deacetylase 5 (HDAC5) kinase, resulting in the nuclear export of HDAC5 and acceleration of transcription of cardiac hypertrophic genes. In addition, GRK5 acts as a positive coregulator of nuclear factor of activated T cells in a kinase‐independent manner to mediate hypertrophic gene transcription [10, 11]. Although various reports have demonstrated the function of GRK5 in cardiac diseases, the role of GRK5 in MI has not yet been elucidated. Moreover, the aforementioned studies focused on the role of GRKs expressed in cardiomyocytes. In the case of MI, nonparenchymal cells play an important role in the formation of cardiac pathology [2, 3, 4], and GRK5 has been reported to be associated with inflammation in nonparenchymal cells [12, 13]. Thus, we examined whether GRK5 contributes to cardiac pathology after MI in nonparenchymal cells.

## Materials and methods

### Ethics statement

Animal experiments were performed in accordance with the relevant national and international guidelines contained in the “Act on Welfare and Management of Animals” (Ministry of Environment of Japan) and “Regulation of Laboratory Animals” (Kyushu University), and all experimental procedures in this study were approved by the Institutional Animal Care and Use Committee at Kyushu University (approval number, A27‐063‐0 and A29‐056‐0).

### Surgical procedure of MI and echocardiographic analysis

We obtained C57BL/6J mice from Charles River Laboratories Japan and Japan SLC, and obtained GRK5 knockout (KO) mice from Dr. RJ Lefkowitz (Duke University). The mice allowed *ad libitum* access to water and food pellets (CE‐2, CLEA Japan, Shizuoka, Japan) and housed in cages (155 W × 245D × 148H mm, CLEA Japan) at 20 °C and 50% humidity under a 12‐h light–dark cycle (light from 8:00 am to 8:00 pm). MI operation and were performed as previously reported [5]. In brief, male mice (8–11 weeks old) were anesthetized and subjected to permanent occlusion of the left coronary artery. Sham‐operated animals underwent the same procedure without occlusion of the coronary artery. The operated mice were allowed time to recover under a warm pad until fully awake. Echocardiographic data were recorded using a Nemio GX image analyzing system (SSA‐580A, Toshiba Medical Systems, Tochigi, Japan). The percent ejection fraction (% EF) was calculated using Teicholz's method. To estimate cardiac systolic function, the percent fractional shortening (% FS) was calculated: % FS = [(LVIDd − LVIDs)/LVIDd] × 100.

### mRNA expression analysis

Total RNA from sham‐ or MI‐operated hearts of mice was extracted using ISOGEN (Nippon Gene, Toyama, Japan) and purified using an RNeasy Mini Kit (Qiagen, Venlo, the Netherlands). Extraction and purification of total RNA from cultured cells were performed using an RNeasy Mini Kit (Qiagen). mRNA expression levels of the indicated genes were quantified using real‐time reverse transcription polymerase chain reaction (RT‐PCR) with dual‐labeled probes. Specific primers and probes used in this study are listed in Table S1.

### Histological analysis

Hearts of wild‐type (WT) or GRK5 KO mice were fixed with 4% paraformaldehyde overnight and embedded in FSC 22 Frozen Section Media (Leica Microsystems, Tokyo, Japan). Cryostat sections (4 μm in thickness) were blocked with 5% bovine serum albumin in phosphate‐buffered saline (PBS) and immunostained with following antibodies: anti‐CD68 (Bio‐Rad Laboratories, Hercules, CA, USA), anti‐α‐smooth muscle actin (SMA; Sigma‐Aldrich, St. Louis, MO, USA), anti‐phospho‐p65 (Cell Signaling Technology, Danvers, MA, USA), anti‐CD31 (BD Biosciences, Franklin Lakes, NJ, USA), and anti‐α‐actinin (Sigma‐Aldrich). Sections were analyzed using fluorescence microscopy (BioRevo BZ‐9000; KEYENCE, Osaka, Japan), and the images obtained were quantified using BZ‐II analyzer (KEYENCE).

### Picrosirius red staining

Paraffin‐embedded heart sections (5 μm in thickness) of WT or GRK5 KO mice were stained with Picrosirius red [3% picric acid (FUJIFILM WAKO Pure Chemical Corporation, Osaka, Japan), 0.15% Direct Red 80 (Sigma‐Aldrich) in distilled water]. The images were captured using microscopy (BioRevo BZ‐9000; KEYENCE). The collagen volume fraction (CVF) of the whole heart was quantified using the BZ‐II analyzer (KEYENCE).

### Cell sorting and extraction of mRNA

Cardiac cells were harvested from the hearts of MI‐operated WT mice hearts as previously reported [5]. The obtained cardiac cells were pretreated with anti‐CD16/32 antibody (BD Biosciences) for 10 min and stained with anti‐CD11b (BioLegend, San Diego, CA, USA) and anti‐platelet‐derived growth factor receptor α (PDGFR‐α) antibodies (BioLegend) for 30 min. The stained cells were washed and sorted using FACSAriaIII (BD Biosciences). After sorting, the cells were placed into ISOGEN (Nippon Gene) and purified using Gene‐Packman Coprecipitant (Nacalai Tesque, Kyoto, Japan) according to the manufacturer's protocol.

### Bone marrow transplantation

Six‐week‐old WT mice were irradiated with 10 Gy from a cesium‐137 gamma source and used as recipient mice. Bone marrow cells were collected from the femurs and tibias of 6‐week‐old WT or GRK5 KO mice and suspended in PBS. The bone marrow cells were transplanted in the recipient mice by injection through the orbital vein. Four weeks after the transplantation, the bone marrow‐transplanted mice were subjected to MI.

### Isolation of cardiac fibroblasts/myofibroblasts and stimulation with TNF‐α

MI‐operated heart ventricles of WT or GRK5 KO mice were minced with surgical knives and digested by shaking in digestion buffer [1 mg·mL^−1^ trypsin (Sigma‐Aldrich), 1 mg·mL^−1^ collagenase A (Roche) in PBS] at 37 °C, and the cells were then collected by centrifugation. These cells were cultured in DMEM supplemented with 10% FBS and 1% penicillin/streptomycin overnight, and the culture medium was changed to remove unattached cells. The attached cells were used as cardiac fibroblasts/myofibroblasts in our experiments. Cardiac fibroblasts/myofibroblasts (1 × 10^5^ cells) were seeded on nontreated six‐well culture plates. One day later, the cells were starved with DMEM supplemented with 0.01% FBS and 1% penicillin/streptomycin for 8 h and then stimulated with mouse TNF‐α (10 ng·mL^−1^, PeproTech, Cranbury, NJ, USA) for the indicated times.

### Western blotting analysis

Cardiac fibroblasts/myofibroblasts were lysed using the lysis buffer [50 mm Tris–HCl (pH 7.5), 150 mm NaCl, 1 mm ethylenediaminetetraacetic acid, 0.5% Nonidet P‐40, 10% glycerol, 20 mm NaF, 1% protease inhibitor cocktail (Nacalai Tesque), 2% PhosSTOP (Roche)] and samples were subjected to western blotting with the following antibodies: anti‐GRK5 [14], anti‐phospho‐p65 (Cell Signaling Technology), anti‐p65 (Cell Signaling Technology), anti‐phospho‐inhibitor of nuclear factor κ light polypeptide gene enhancer in B‐cells inhibitor, α (IκBα; Cell Signaling Technology), anti‐IκBα (Cell Signaling Technology), and anti‐GAPDH (Santa Cruz Biotechnology). As a secondary antibody, anti‐rabbit IgG (Cell Signaling Technology), or anti‐mouse IgG (Santa Cruz Biotechnology) conjugated with horseradish peroxidase was used. Densitometric analysis was conducted using the ImageJ software (National Institutes of Health).

### Statistical analysis

The results were presented as mean ± standard error of mean of at least three independent experiments and analyzed using unpaired two‐tailed Student's *t*‐test and Mann–Whitney *U* test for comparison of the two groups or one‐way analysis of variance using the Newman–Keuls method for multiple group comparison.

## Results

### GRK5 expression is upregulated in the hearts after MI

**Fig. 1:**
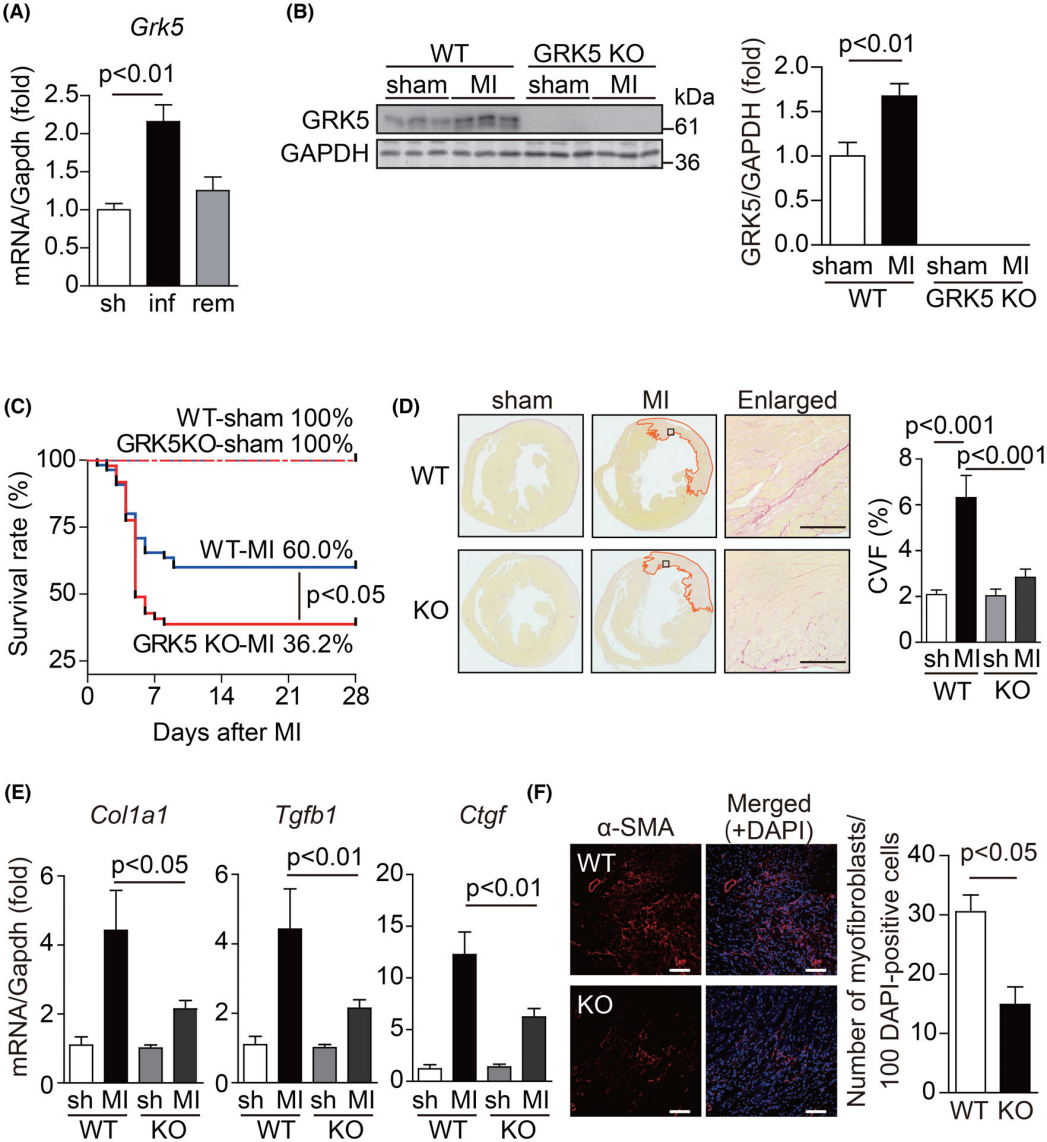
GRK5 deficiency leads to high mortality after MI because of reduced fibrosis. (A) The mRNA expression levels of GRK5 3 days after MI in mouse hearts. Sham‐operated heart was used as a control; “sh” represents sham, “inf” and “rem” represent the infarcted and remote areas of the MI‐operated heart, respectively; *n* = 5–6. (B) GRK5 protein expression levels in WT hearts and GRK5 KO hearts 4 days after sham or MI operation. Samples were subjected to western blotting using anti‐GRK5 and anti‐GAPDH antibody. Quantitative data of the western blotting are shown in the right panel. (C) Kaplan–Meier survival curves of WT and GRK5 KO after sham or MI operation. The difference between WT‐MI and GRK5 KO‐MI was evaluated by the log‐rank test (*P* < 0.05). WT‐sham: *n* = 15; WT‐MI: *n* = 55; GRK5 KO‐sham: *n* = 10; GRK5 KO‐MI: *n* = 48. (D) Picrosirius red staining of the ventricular sections from WT and GRK5 KO mice 4 days after MI and sham operation. The area surrounded by the orange line indicates the infarct region of MI. The squares in the infarct area of MI panels are magnified in the enlarged panels. Quantitative data of collagen volume fraction (CVF) are shown in the right panel; *n* = 3. Scale bar = 100 μm. (E) mRNA expression levels of fibrosis‐related genes in the hearts of WT and GRK5 KO mice 3 days after MI. Total RNA extracted from sham‐ or MI‐operated ventricles was subjected to real‐time RT‐PCR. *Gapdh* was used as an internal control; *n* = 5. (F) Heart section of WT and GRK5 KO mice 3 days after MI was stained with anti‐α‐SMA antibody. Representative images of each infarct area are shown. Quantitative data of α‐SMA‐positive myofibroblasts are shown in the right panel; *n* = 3–4. Scale bar = 50 μm. Statistical analysis was performed using one‐way analysis of variance with Newman–Keuls multiple comparison test (A, B, D, and E) and unpaired two‐tailed Student's *t*‐test (F). Error bars represent the mean ± SEM.

We first examined the mRNA expression level of GRK5 in the hearts of mice 3 days after sham or MI operation. GRK5 mRNA levels substantially increased in the infarcted area of hearts after MI operation (Fig. 1A). Consistently, the protein expression level of GRK5 also significantly increased after MI (Fig. 1B), suggesting that GRK5 is involved in the pathogenesis of MI.

### GRK5 deficiency leads to high mortality after MI

To examine the role of GRK5 in MI, we compared the survival rate of WT mice and GRK5 KO mice for 28 days after MI. Comparison of the survival rate after MI demonstrated that the survival rate of GRK5 KO mice (17/47 = 36.2%) at day 28 after MI significantly decreased compared to that of WT mice (33/55 = 60.0%; Fig. 1C). The mortality rate was particularly high among the GRK5 KO mice from day 4 to day 7 after MI, while no difference in mortality rate was observed in the mid‐ to late post‐MI period. Echocardiographic analysis revealed that there were no differences in cardiac function between sham‐operated WT and GRK5 KO mice (Fig. S1), as previously reported [9]. This result indicated that GRK5 deficiency in cardiac cells under normal conditions does not significantly influence cardiac functions. Similarly, among the MI‐operated mice, no significant difference in cardiac function was observed between WT and GRK5 KO mice on Day 28 post‐MI (Fig. S1). These results suggested that GRK5 plays a cardioprotective role in the early phase of MI.

### GRK5 promotes fibrosis after MI

Next, we performed pathological analysis to examine differences in the survival ratio between WT and GRK5 KO mice after MI. We performed Picrosirius red staining of the heart sections of WT and GRK5 KO mice 4 days after MI to evaluate the degree of fibrosis. The degree of fibrosis was found to be substantially higher in the infarcted hearts of WT mice (Fig. 1D). However, the promotion of fibrosis after MI was significantly attenuated in GRK5 KO mice (Fig. 1D). To confirm this result, we measured the mRNA expression levels of fibrosis‐related genes, such as *Col1a1*, *Tgfb1*, and *Ctgf* in mouse hearts 3 days after MI using real‐time RT‐PCR. We found that their levels in GRK5 KO mice were lower than those in WT mice (Fig. 1E). These fibrotic proteins were produced in myofibroblasts. Myofibroblasts, which are not present in normal hearts, emerge in the infarcted area after MI and mediate fibrosis by producing many extracellular matrix proteins [2, 3, 4, 5, 15, 16]. Thus, we proceeded to compare the number of myofibroblasts at the infarcted area of WT and GRK5 KO mice. Immunohistochemical analysis showed that the number of myofibroblasts positive for α‐SMA, a representative marker for myofibroblasts, in GRK5 KO hearts was around 50% lower than that in WT hearts (Fig. 1F). These data show that GRK5 deficiency attenuates fibrosis after MI. Insufficient fibrosis leads to cardiac rupture after MI [4, 5], suggesting that susceptibility to cardiac rupture may be a factor responsible for the increased mortality in GRK5 KO mice.

### GRK5 enhances inflammatory responses after MI via activation of NF‐κB signaling

**Fig. 2:**
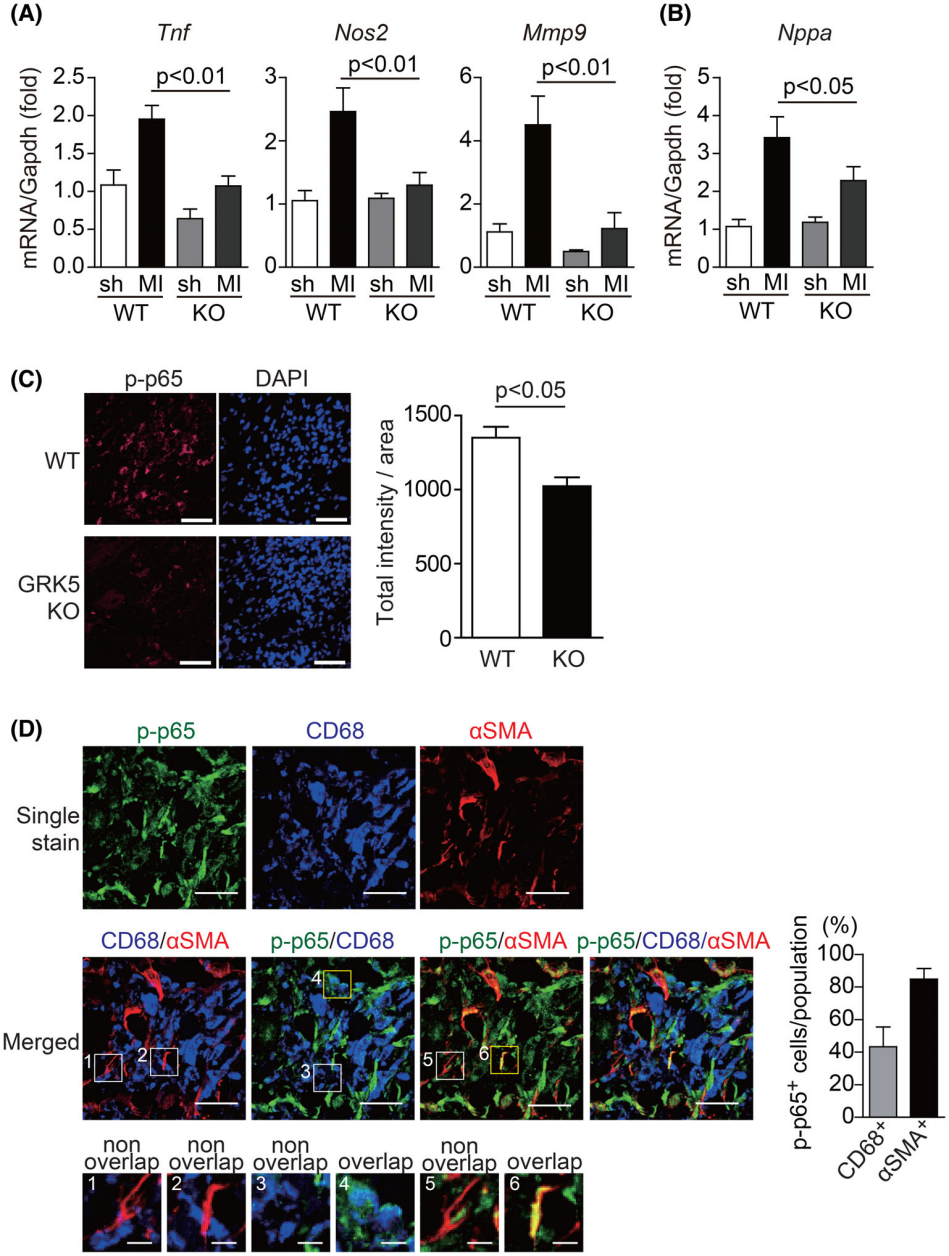
GRK5 enhances inflammatory responses after MI via the activation of NF‐κB signaling. (A, B) mRNA expression levels of inflammation‐related genes (A) and a hypertrophic gene, *Nppa*, (B) in hearts of WT and GRK5 KO mice 3 days after MI. Total RNA extracted from sham‐ or MI‐operated ventricles was subjected to real‐time RT‐PCR. *Gapdh* was used as an internal control; *n* = 5. sh, sham. (C) Heart sections of WT and GRK5 KO mice 3 days after MI were stained with anti‐phospho‐p65 (Ser536) antibody. Representative results are shown. Quantitative data are shown in right panel; *n* = 3–4; Scale bar = 50 μm. (D) Heart section of WT mice 3 days after MI was stained with anti‐phospho‐p65 (Ser536; green), anti‐CD68 (blue), and anti‐α‐SMA (red) antibody. CD68 and α‐SMA were used as markers of macrophages and myofibroblasts, respectively. The area indicated by white squares (single‐positive cells) and yellow squares (double‐positive cells) on merged images were enlarged, respectively. The percentage of p‐p65‐positive cells in CD68‐positive, αSMA‐positive cells is shown in a bar graph; *n* = 4; Scale bar = 25 μm (lower magnification), 5 μm (higher magnification). Statistical analysis was performed using one‐way analysis of variance using Newman–Keuls multiple comparison test (A and B) and Mann–Whitney *U* test (C). Error bars represent the mean ± SEM.

It is known that inflammation triggers fibrosis [2, 3, 4, 5]. Therefore, we next investigated inflammatory responses in the infarcted hearts of WT and GRK5 KO mice. Real‐time RT‐PCR revealed that the mRNA expression levels of inflammation‐related genes such as TNF‐α (*Tnf*), *Nos2*, and *Mmp9* were markedly lower in GRK5 KO mice than in WT mice 3 days after MI (Fig. 2A). We also measured the expression level of a cardiac hypertrophic gene, atrial natriuretic peptide (*Nppa*), in infarcted hearts and found that *Nppa* was induced by MI operation. The induction of *Nppa* by MI operation was significantly attenuated in GRK5 KO mouse hearts (Fig. 2B), which is consistent with previous reports stating that GRK5 promotes cardiac hypertrophic genes in cardiomyocytes [8, 9].

Nuclear factor (NF)‐κB signaling is one mechanism that regulates inflammatory gene transcription [17], and it has been reported that GRK5 and GRK6 (which belongs to the same subfamily as GRK5) directly phosphorylate IκBα and regulate NF‐κB signaling [12, 18, 19]. We thus examined phosphorylation of p65, a component of NF‐κB [18, 20], in WT or GRK5 KO infarcted hearts to observe the activation of NF‐κB signaling. Immunohistochemical analysis showed that the content of phosphorylated p65 reduced in GRK5 KO hearts after MI compared to that in WT hearts 3 days after MI (Fig. 2C). Further analysis revealed phosphorylated p65 signals in both macrophages (CD68‐positive cells) and myofibroblasts (α‐SMA‐positive cells) in MI‐operated WT mice hearts (Fig. 2D). By contrast, phosphorylated p65 signals were not detected in CD31‐positive endothelial cells (Fig. S2A) and α‐actinin‐positive cardiomyocytes (Fig. S2B). These data suggest that around 40% of macrophages and about 80% of myofibroblasts were involved in inflammation via the activation of NF‐κB signaling 3 days after MI.

### GRK5 expressed in bone marrow cells contributes insignificantly to inflammatory responses after MI

**Fig. 3:**
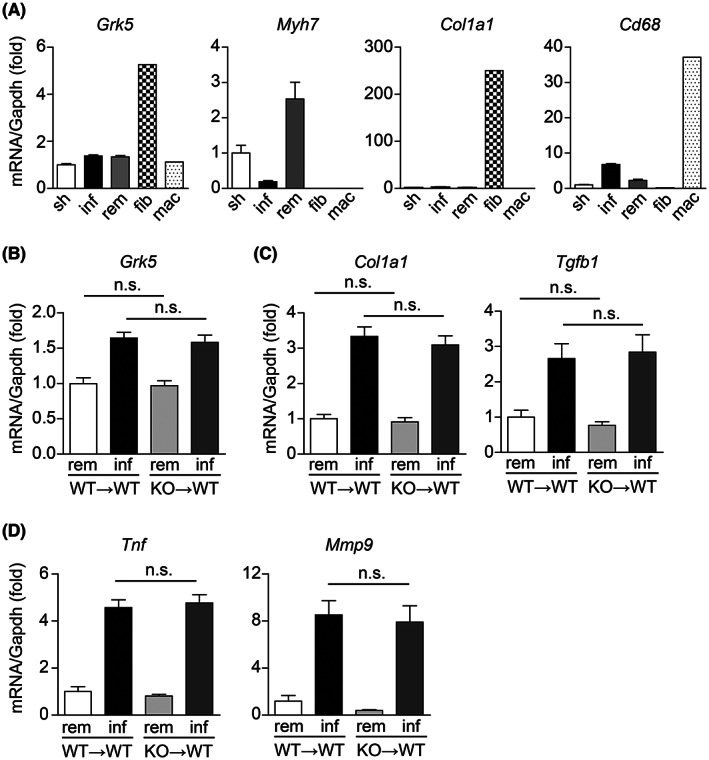
GRK5 expressed in bone marrow cells does not contribute to inflammatory responses after MI. (A) mRNA expression levels of the indicated genes 3 days after MI in sorted fibroblasts/myofibroblasts (fib) and macrophages (mac) as well as sham (sh) and MI‐operated hearts. Infarcted mouse hearts were subdivided into infarct area (inf) and noninfarct remote area (rem). *Myh7*, *Col1a1*, and *Cd68* were used as markers for cardiomyocytes, myofibroblasts, and macrophages, respectively. (B–D) Bone marrow transfer experiments. WT mice were transplanted with bone marrow cells from WT (WT → WT) or GRK5 KO (KO → WT) mice and then treated for MI. Total RNA extracted from infarcted and remote areas in MI‐operated hearts were subjected to real‐time RT‐PCR. mRNA expression levels of (B) GRK5, (C) fibrosis‐related genes, and (D) inflammation‐related genes 3 days after MI. WT → WT; *n* = 5, KO → WT; *n* = 6. Statistical analysis was performed using one‐way analysis of variance with Newman–Keuls multiple comparison test. Error bars represent the mean ± SEM (n.s., not significant).

To examine whether GRK5 contributes to the inflammation regulated by NF‐κB signaling, we next investigated the expression level of GRK5 in macrophages and cardiac fibroblasts to determine the cell types that express GRK5. We digested mouse hearts 3 days after MI and subsequently stained the isolated cardiac cells with anti‐CD11b (a macrophage marker) and anti‐PDGFR‐α (a marker of fibroblasts/myofibroblasts) antibodies to collect macrophages and cardiac fibroblasts/myofibroblasts using a cell sorter (Fig. S3). Real‐time RT‐PCR analysis showed that *Cd68* (a marker of macrophages) and *Col1a1* (a marker of myofibroblasts) were only detected in CD11b‐positive and PDGFR‐α‐positive fractions, respectively, and that *Myh7* (a marker of cardiomyocytes [21, 22]) was hardly detected in the CD11b‐positive or PDGFR‐α‐positive fraction (Fig. 3A). Therefore, the purity and quality of the collected macrophages and fibroblasts/myofibroblasts were considered to be sufficient. We found that GRK5 was highly expressed in fibroblasts/myofibroblasts compared to that in macrophages (Fig. 3A), suggesting that GRK5 contributes to inflammation mainly in fibroblasts/myofibroblasts. To further clarify the influence of GRK5 on inflammation via cardiac macrophages, we performed a bone marrow transfer experiment. Lethally irradiated WT mice were reconstituted with bone marrow cells from WT or GRK5 KO mice. The mRNA expression levels of GRK5 in MI‐operated mouse hearts were almost the same between WT mice transplanted with bone marrow cells from WT mice (WT → WT) and WT mice transplanted with bone marrow cells from KO mice (KO → WT) (Fig. 3B). This result suggests that the expression level of GRK5 was insignificant in bone marrow‐derived cells. Consistent with this result, there were no significant changes in the expression of fibrosis‐related (Fig. 3C) and inflammatory genes (Fig. 3D) between WT → WT and KO → WT mice. These results further show that GRK5 in fibroblasts/myofibroblasts, but not in bone marrow‐derived cells, mainly contributes to inflammatory responses in the infarcted hearts.

### GRK5 in cardiac fibroblasts/myofibroblasts enhances inflammatory responses via NF‐κB activation

**Fig. 4:**
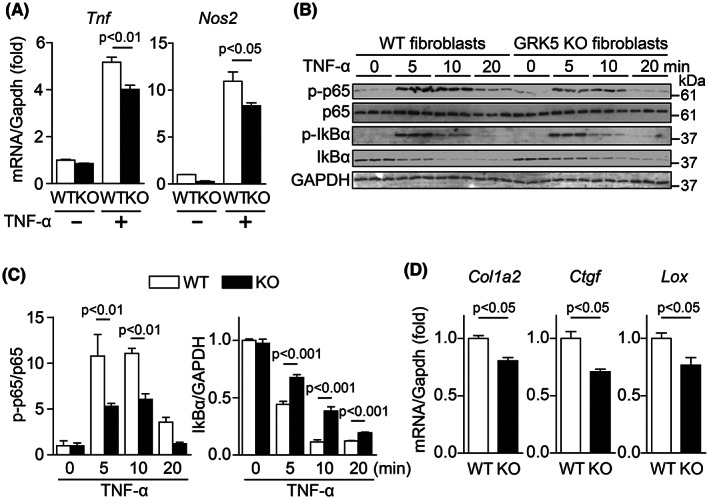
GRK5 in fibroblasts/myofibroblasts enhances inflammatory responses via NF‐κB activation. (A) mRNA expression levels of inflammation‐related genes in cardiac fibroblasts/myofibroblasts of WT and GRK5 KO after TNF‐α stimulation. Cardiac fibroblasts/myofibroblasts isolated from heart 3 days after MI were serum‐starved for 8 h and then stimulated with TNF‐α (10 ng·mL^−1^) for 24 h. The samples were subjected to real‐time RT‐PCR. The data of WT and GRK5 KO mice are shown in white and black columns, respectively; *n* = 3. (B) Cardiac fibroblasts/myofibroblasts isolated from heart 3 days after MI were serum‐starved for 8 h and then stimulated with TNF‐α (10 ng·mL^−1^) for indicated times. Samples were subjected to western blotting using anti‐p‐p65, anti‐p65, anti‐p‐IκBα, anti‐IκBα and anti‐GAPDH antibody. (C) Quantitative data of western blotting; p‐p65 and IκBα were normalized with p65 and GAPDH, respectively. The data of WT and GRK5 KO cardiac fibroblasts/myofibroblasts are shown in white and black columns, respectively; *n* = 3. (D) mRNA expression levels of fibrosis‐related genes in WT and GRK5 KO cardiac fibroblasts/myofibroblasts of the samples prepared in Fig. 4A. Statistical analysis was performed using one‐way analysis of variance with Newman–Keuls multiple comparison test (A and C) and Mann–Whitney *U* test (D). Error bars represent the mean ± SEM.

Our results and reports demonstrating that cardiac fibroblasts/myofibroblasts contribute to inflammation after MI [23] prompted us to further examine whether GRK5 in cardiac fibroblasts/myofibroblasts promotes inflammation at the infarcted area. We isolated cardiac fibroblasts/myofibroblasts from hearts of WT or GRK5 KO mice 3 days after MI. TNF‐α stimulation of the isolated WT cardiac fibroblasts/myofibroblasts induced the expression of inflammatory genes such as *Tnf* and *Nos2* (Fig. 4A). However, this induction was significantly attenuated in GRK5 KO cardiac fibroblasts/myofibroblasts (Fig. 4A). Consistent with this result, TNF‐α‐induced phosphorylation of p65 and IκBα was suppressed in cardiac fibroblasts/myofibroblasts isolated in GRK5 KO mice. In addition, the degradation of the total amount of IκBα was suppressed in GRK5 KO fibroblasts/myofibroblasts (Fig. 4B,C). These data suggest that GRK5 expressed in cardiac fibroblasts/myofibroblasts enhanced inflammatory responses after MI via the activation of NF‐κB signaling. Finally, we examined whether the activation of NF‐κB signaling by TNF‐α stimulation in fibroblasts/myofibroblasts alters the expression levels of fibrosis‐related genes. Expression levels of *Col1a2*, *Ctgf*, and *Lox* were significantly suppressed in GRK5 KO fibroblasts/myofibroblasts (Fig. 4D). This result suggested that GRK5 promotes fibrosis by regulating the differentiation of fibroblasts into myofibroblasts. Taken together, GRK5, expressed in fibroblasts/myofibroblasts in the early stage of MI, may contribute to cardioprotection from lethal heart damage by enhancing inflammation and fibrosis.

## Discussion

Cytokines such as TNF‐α and TGF‐β1 have been shown to promote the differentiation of fibroblasts into myofibroblasts and promote fibrosis through NF‐κB activation in animal models of fibrosis, such as bleomycin‐induced pulmonary fibrosis [24] and unilateral ureteral obstruction (UUO)‐induced renal fibrosis [25]. Consistent with these previous reports, this study revealed that TNF‐α stimulation enhances NF‐κB activation in cardiac fibroblasts/myofibroblasts and that GRK5 mediates the inflammation via the TNF‐α/ NF‐κB signaling pathway and promotes fibrosis in MI. In addition to the involvement of GRK5 in the TNF‐α/ NF‐κB signaling pathway in cardiac myofibroblasts, we also showed that the mRNA expression levels of collagens were significantly attenuated by TNF‐α stimulation in GRK5 KO cardiac fibroblasts/myofibroblasts compared with that in WT mice (Fig. 4D). In fact, the number of αSMA‐positive myofibroblasts significantly decreased in the infarcted area of GRK5 KO mice (Fig. 1F). These results suggested that GRK5 is involved not only in fibrosis by promoting inflammation but also in the differentiation of fibroblasts into myofibroblasts. GRK5 is reportedly involved in various inflammatory pathologies through GPCR‐ and non‐GPCR‐mediated pathways [26, 27, 28, 29]. We reported that GRK5 is involved in NF‐κB signaling activation in fibroblasts/myofibroblasts using TNF‐α, the level of which is substantially increased in the infarcted area [23]. Therefore, GRK5 in cardiac fibroblasts/myofibroblasts is likely activated through the non‐GPCR‐mediated pathway.

In this study, we focused on the early stage of MI because the survival rate of GRK5 KO mice was significantly lower than that of WT mice in this period (Fig. 1C). Cardiomyocyte death via apoptosis and necrosis is particularly increased in the early stage of MI, and insufficient fibrosis during this period promotes cardiac rupture [30, 31]. Therefore, we consider that GRK5 exerts beneficial effects on the heart, such as preventing cardiac rupture by promoting inflammation and fibrosis. However, long‐term inflammation and fibrosis have been shown to promote cardiac remodeling and worsen cardiac functions [32]. Very recently, mice with fibroblast‐specific deletion of GRK5 were reported to have reduced cardiac remodeling during the late stages of MI [26]. In addition, cardiomyocyte‐specific GRK5‐deficient mice reduced immune cell infiltration and inflammation during MI, leading to suppression of fibrosis [33]. Considering these reports, the extreme suppression of fibrosis in MI‐operated GRK5 KO mice in this study may be due to the lack of both the profibrotic and pro‐inflammatory effects of GRK5 on fibroblasts and cardiomyocyte, respectively. As a result, GRK5 KO mice would be more susceptible to cardiac rupture and have higher mortality due to severe suppression of fibrosis in the acute phase of MI.

In recent years, the incidence of cardiac rupture following MI has been decreasing due to increased use of reperfusion therapies, better control of blood pressure, and use of medicines such as β‐blockers, angiotensin‐converting enzyme inhibitors, and anticoagulants [34]. However, since the occurrence of cardiac rupture is a lethal condition, it is extremely important to prevent it. We demonstrated that GRK5 is a key molecule that confers protection to heart tissue in the acute phase of MI. On the contrary, fibroblast‐specific GRK5 KO mice showed reduced fibrosis and improved cardiac function at 4‐week post‐MI [26], implying that GRK5 in fibroblasts/myofibroblasts is a risk molecule that worsens cardiac function in the late phase of MI. Since increased GRK5 expression at the infarct area was still observed at 28 days post‐MI (Fig. S4), GRK5 is likely continuously activated and involved in the pathology of the late phase of MI. Conventional GRK5 KO mice in this study did not show improved cardiac function compared with WT mice at 28 days post‐MI (Fig. S1), suggesting that cardiac cells, excluding fibroblasts/myofibroblasts, may be involved in cardioprotection against MI. One possibility is that aldosterone and the mineralocorticoid receptor, whose expression increases after MI, promote inflammation and fibrosis of the infarcted area [35], and that GRK5 expression by cardiomyocytes inhibits the aldosterone‐mineralocorticoid signaling pathway [36]. Taken together, these results suggest that the long‐term upregulation of GRK5 expression in cardiomyocytes may have beneficial effects on aldosterone‐associated inflammation and fibrosis; therefore, the appropriate regulation of GRK5 activity is required if GRK5 is a therapeutic target for MI.

## Conflict of interest

The authors declare no conflict of interest.

## Author contributions

AN, TT, MNo, and MT performed some essential *in vivo* and *in vitro* experiments and wrote the paper. MF and YY performed some experiments. SA and TK provided key resources and contributed to the scientific discussion. MNa conceived the project, writing‐reviewing and editing, supervision. All authors reviewed and approved the final version of the manuscript for submission.

## Supporting information


**Fig. S1** Echocardiography of WT mice and GRK5 KO mice 28 days after MI or sham operation. (A) Representative images of M‐mode echocardiography of sham‐ or MI‐ operated WT and GRK5 KO mice on day 28 day after operation. LVIDd, Left ventricular end diastolic internal diameter; LVIDs, Left ventricular end systolic internal diameter. (B) Sham‐ or MI‐ operated WT and GRK5 KO mice on day 28 after operation were evaluated for their cardiac function by measuring the ejection fraction (EF) and fractional shortening (FS). The echocardiogram revealed that both EF and FS were comparable between WT and GRK5 KO mice. WT‐sham: n=5; WT‐MI: n=19; GRK5 KO‐sham: n=11; GRK5 KO‐MI: n=13. Statistical analysis was performed using one‐way analysis of variance with Newman‐Keuls multiple comparison test. Error bars represent the mean ±SEM (n.s., not significant).
**Fig. S2.** Phosphorylated‐p65 signals could not be observed in endothelial cells and cardiomyocytes after MI. Heart section of WT mice at 3 days after MI was stained with anti‐p‐p65 (red) and anti‐CD31 (green) antibody (A) or anti‐α‐actinin (green) antibody (B). CD31 and anti‐α‐actinin are markers of endothelial cells and cardiomyocytes, respectively. The area indicated by yellow squares on the merged images were enlarged. Scale bar is 50 μm.
**Fig. S3.** Gating strategy for isolating cardiac cells. Isolated cells from hearts at 3 days after MI were stained with Viability dye, anti‐PDGFR‐α and anti‐CD11b antibody. The cells were gated for selecting living cells and singlets, and further gated at PDGFR‐α+ cells (fibroblasts/myofibroblasts) and CD11b+ cells (macrophages). mRNA was extracted from each cell population.
**Fig. S4**. GRK5 expression in infarct area remains high on post‐MI day 28. GRK5 mRNA expression levels in infarct area (inf) and remote area (rem) of MI‐operated WT mice were measured by real time RT‐PCR. Sham‐operated heart (sh) was used as a control; sham n=3, MI n=7. Statistical analysis was performed using one‐way analysis of variance with Newman‐Keuls multiple comparision test. Error bars represent the mean ±SEM (n.s., not significant).

**Table S1** Sequences of primers of Assay ID used for real‐time RT‐PCR.

## Data Availability

The data that support the findings of this study can be obtained from the corresponding author on reasonable request.
