# Supplementary material for: GRK5‐mediated inflammation and fibrosis exert cardioprotective effects during the acute phase of myocardial infarction
Source: FEBS Open Bio. 2023 Jan 20;13(2):380–91. doi: 10.1002/2211-5463.13551 (PMC9900089; doi:10.1002/2211-5463.13551)
Supplement: Supplementary file 1 — Fig. S1 Echocardiography of WT mice and GRK5 KO mice 28 days after MI or sham operation. (A) Representative images of M‐mode echocardiography of sham‐ or MI‐ operated WT and GRK5 KO mice on day 28 day after operation. LVIDd, Left ventricular end diastolic internal diameter; LVIDs, Left ventricular end systolic internal diameter. (B) Sham‐ or MI‐ operated WT and GRK5 KO mice on day 28 after operation were evaluated for their cardiac function by measuring the ejection fraction (EF) and fractional shortening (FS). The echocardiogram revealed that both EF and FS were comparable between WT and GRK5 KO mice. WT‐sham: n=5; WT‐MI: n=19; GRK5 KO‐sham: n=11; GRK5 KO‐MI: n=13. Statistical analysis was performed using one‐way analysis of variance with Newman‐Keuls multiple comparison test. Error bars represent the mean ±SEM (n.s., not significant). Fig. S2. Phosphorylated‐p65 signals could not be observed in endothelial cells and cardiomyocytes after MI. Heart section of WT mice at 3 days after MI was stained with anti‐p‐p65 (red) and anti‐CD31 (green) antibody (A) or anti‐α‐actinin (green) antibody (B). CD31 and anti‐α‐actinin are markers of endothelial cells and cardiomyocytes, respectively. The area indicated by yellow squares on the merged images were enlarged. Scale bar is 50 μm. Fig. S3. Gating strategy for isolating cardiac cells. Isolated cells from hearts at 3 days after MI were stained with Viability dye, anti‐PDGFR‐α and anti‐CD11b antibody. The cells were gated for selecting living cells and singlets, and further gated at PDGFR‐α+ cells (fibroblasts/myofibroblasts) and CD11b+ cells (macrophages). mRNA was extracted from each cell population. Fig. S4. GRK5 expression in infarct area remains high on post‐MI day 28. GRK5 mRNA expression levels in infarct area (inf) and remote area (rem) of MI‐operated WT mice were measured by real time RT‐PCR. Sham‐operated heart (sh) was used as a control; sham n=3, MI n=7. Statistical analysis was performed using one‐way analysis of [file FEB4-13-380-s001.pdf]

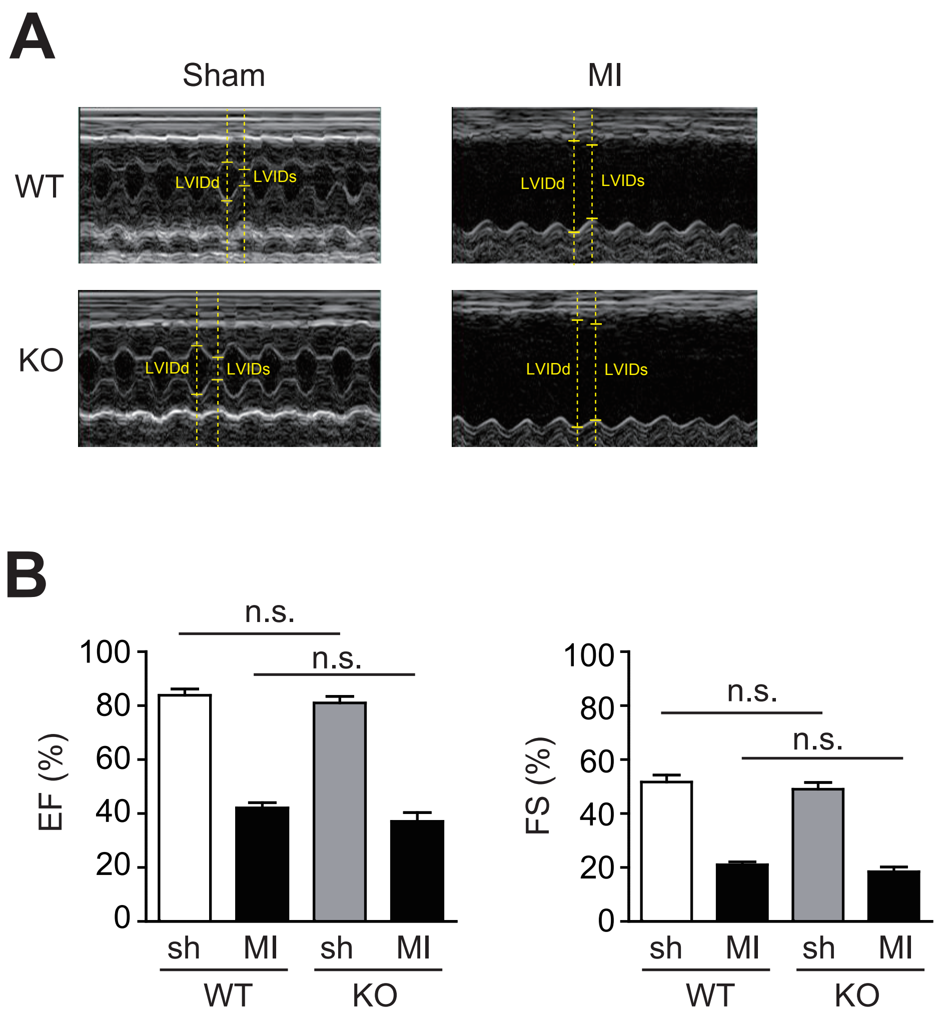

Supplemental Figure 1. Echocardiography of WT mice and GRK5 KO mice 28 days after MI or sham operation.

(A) Representative images of M-mode echocardiography of sham- or MI- operated WT and GRK5 KO mice on day 28 day after operation. LVIDd, Left ventricular end diastolic internal diameter; LVIDs, Left ventricular end systolic internal diameter.

(B) Sham- or MI- operated WT and GRK5 KO mice on day 28 after operation were evaluated for their cardiac function by measuring the ejection fraction (EF) and fractional shortening (FS). The echocardiogram revealed that both EF and FS were comparable between WT and GRK5 KO mice. WT-sham: n=5; WT-MI: n=19; GRK5 KO-sham: n=11; GRK5 KO-MI: n=13. Statistical analysis was performed using one-way analysis of variance with Newman-Keuls multiple comparison test. Error bars represent the mean ±SEM (n.s., not significant).

Supplemental Figure 1

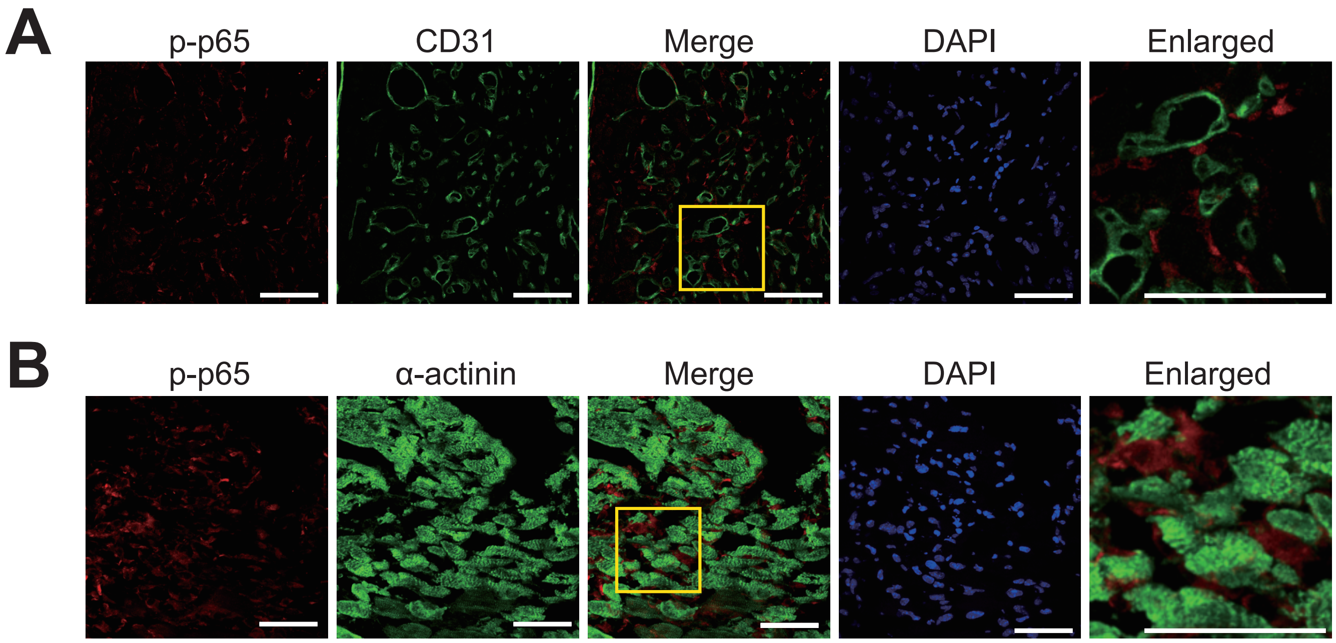

Supplemental Figure 2. Phosphorylated-p65 signals could not be observed in endothelial cells and cardiomyocytes after MI.
Heart section of WT mice at 3 days after MI was stained with anti-p-p65 (red) and anti-CD31 (green) antibody (A) or anti-α-actinin (green) antibody (B). CD31 and anti-α-actinin are markers of endothelial cells and cardiomyocytes, respectively. The area indicated by yellow squares on the merged images were enlarged. Scale bar is 50 μm.

Supplemental Figure 2

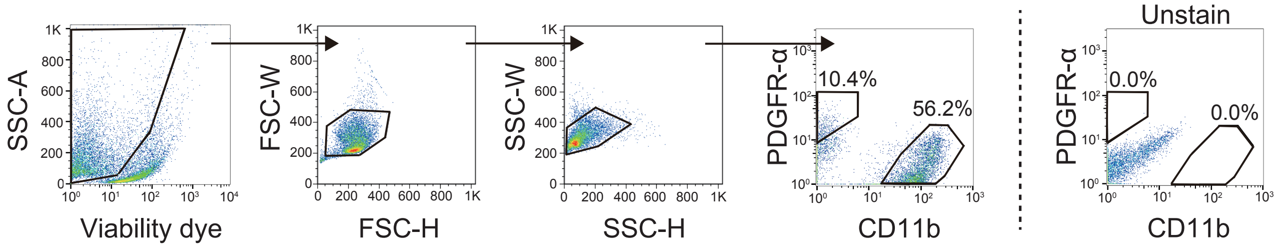

Supplemental Figure 3. Gating strategy for isolating cardiac cells.

Isolated cells from hearts at 3 days after MI were stained with Viability dye, anti-PDGFR-α and anti-CD11b antibody. The cells were gated for selecting living cells and singlets, and further gated at PDGFR-α^{+} cells (fibroblasts/myofibroblasts) and CD11b^{+} cells (macrophages). mRNA was extracted from each cell population.

Supplemental Figure 3

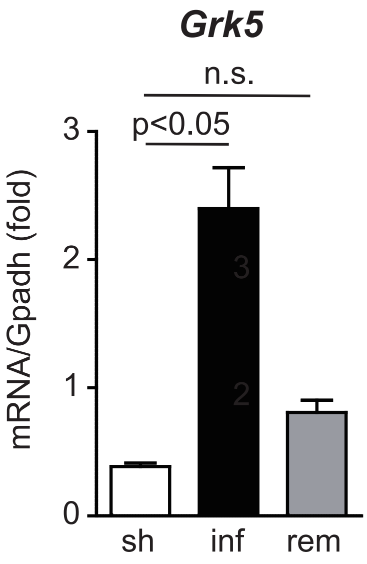

Supplemental Figure 4. GRK5 expression in infarct area remains high on post-MI day 28. GRK5 mRNA expression levels in infarct area (inf) and remote area (rem) of MI-operated WT mice were measured by real time RT-PCR. Sham-operated heart (sh) was used as a control; sham n=3, MI n=7. Statistical analysis was performed using one-way analysis of variance with Newman-Keuls multiple comparision test. Error bars represent the mean ±SEM (n.s., not significant).

Supplemental Figure 4
