# Supplementary material for: GRK5‐mediated inflammation and fibrosis exert cardioprotective effects during the acute phase of myocardial infarction
Source: FEBS Open Bio. 2023 Jan 20;13(2):380–91. doi: 10.1002/2211-5463.13551 (PMC9900089; doi:10.1002/2211-5463.13551)
Supplement: Supplementary file 2 — Table S1 Sequences of primers of Assay ID used for real‐time RT‐PCR. [file FEB4-13-380-s002.pdf]

Supplemental Table 1. Sequences of primers of Assay ID used for real time RT-PCR

mRNA	Sequences of primers (Sigma) or Assay ID (Thermo Fisher)
Col1a2	Forward: 5'-CAAGCATGTCTGGTTAGGAGAGA-3'
	Reverse: 5'-GCTGAGTTGCCATTTCCTTGG-3'
	Probe: 5'- FAM-CCCTTCTACGTTGTATTCAAACTGGCTGCC-TAMRA -3'
Ctgf	Forward: 5'-GGACCGCACAGCAGTTGG-3'
	Reverse: 5'-GGCAGTTGGCTCGCATCATA-3'
	Probe: 5'- FAM-TTTCCCTAGCTGCCTACCGACTGGAAGACACATT-TAMRA -3'
Gapdh	Forward: 5'-CGTCCCGTAGACAAAATGGTGA-3'
	Reverse: 5'-CCACTTTGCCACTGCAAATGG-3'
	Probe: 5'- FAM-CCAATACGGCCAAATCCGTTCACACCGA-TAMRA -3'
Grk5	Forward: 5'-GATTACTACAGTCTATGTGACAAGCA-3'
	Reverse: 5'-CTAAGTCCAGGAACTGAATGTAGC-3'
	Probe: 5'- FAM-TTTCGACAGTTCTGTGAAACCAGGCC-TAMRA -3'
Lox	Forward: 5'-CAAGCTGGTTTCTCGCCGTC-3'
	Reverse: 5'-TGTAGGGGTCGTCGCCCA-3'
	Probe: 5'- FAM-ATCGCCACAGCCTCCGCAGCTCAG-TAMRA -3'
Cd68	Mm03047340_m1
Col1a1	Mm00801666_g1
Ctgf	Mm01192932_g1
Gapdh	Mm99999915_g1
Mmp9	Mm00600163_m1
Myh7	Mm01319006_g1
Nos2	Mm00440502_m1
Nppa	Mm01255747_g1
Tgfb1	Mm01178820_m1
Tnf	Mm00443258_m1

Supplemental Table 1
